# Keystone Veterinary Medical Association

**Published:** 1894-11

**Authors:** W. L. Rhoads

**Affiliations:** Secretary


### KEYSTONE VETERINARY MEDICAL ASSOCIATION.

The Keystone Veterinary Medical Association held its regular monthly meeting at the Philadelphia Veterinary Sanitarium on Tuesday evening, November 13th. President Lintz in the chair. On roll call by the Secretary, Dr. Rhoads, Drs. Bridge, Cullen, Lintz, Rhoads, Hoskins, Schrieber, Jas. B. Rayner and Jno. R. Hart answered to their names.

Drs. F. S. Allen and Jas. T. McAnulty were present as visitors.

The following members were named as a Board of Directors to serve during the ensuing year: Drs. Hoskins, Rhoads, Goentner, Bridge and Jno. R. Hart.

The evening was spent in listening to a paper read by Dr. Hoskins, entitled "How might we best spend the winter sessions of our Association?" This was afterward freely discussed.

The action of the City Board of Health in demanding that after a sixty days' notification to producers of milk they shall furnish to said Board a certificate showing the condition of their cattle and their freedom from tuberculosis, based upon a tuberculin test, was freely discussed in its various phases. It was very much regretted by the members that the bill proposing to appropriate a certain sum of money for this purpose had been defeated by the last session of the State Legislature, and this last movement upon the City Board of Health left the farmers without any compensation for their loss, and fixed upon them the cost of the professional work attendant upon making such an examination.

A number of interesting cases were reported, and the subject of Puerpera Hemorrhagica was incidentally discussed in connection with the case which was exhibited to the members in attendance.

Dr. Jas. B. Rayner was appointed to prepare a report upon cases for the December meeting, and Dr. A. F. Schrieber a short paper covering the duties relative to the position which he holds as inspector of meats for the Department of Public Safety of Philadelphia.

W. L. Rhoads, *Secretary.*

---

The December meeting of the Veterinary Medical Association of New Jersey will be held at the State House, Trenton, on December 13th, and we have no doubt that there will be given thorough consideration to many matters of great importance to the veterinary world at that time. We understand that a more modified bill in regard to bovine tuberculosis will be under consideration with a view of ranging New Jersey in line with many of the other States in dealing with this important subject.

